# Similar eicosapentaenoic acid and docosahexaenoic acid plasma levels achieved with fish oil or krill oil in a randomized double-blind four-week bioavailability study

**DOI:** 10.1186/s12944-015-0109-z

**Published:** 2015-09-02

**Authors:** Karin Yurko-Mauro, Jaroslav Kralovec, Eileen Bailey-Hall, Vanessa Smeberg, Jeffrey G. Stark, Norman Salem

**Affiliations:** DSM Nutritional Products, Columbia, MD USA; DSM Nutritional Products, Dartmouth, NS USA; Worldwide Clinical Trials, San Antonio, TX USA

**Keywords:** Docosahexaenoic acid, Eicosapentaenoic acid, Krill, Plasma, Red blood cell, Fish oil, Omega-3, Bioavailability, Bioequivalence

## Abstract

**Background:**

Long-chain n-3 polyunsaturated fatty acids (LC n-3-PUFA), docosahexaenoic acid (DHA) and eicosapentaenoic acid (EPA) provide multiple health benefits for heart, brain and eyes. However, consumption of fatty fish, the main source of LC n-3-PUFAs is low in Western countries. Intakes of LC n-3-PUFA can be increased by taking dietary supplements, such as fish oil, algal oil, or krill oil. Recently, conflicting information was published on the relative bioavailability of these omega-3 supplements. A few studies suggested that the phospholipid form (krill) is better absorbed than the fish oil ethyl ester (EE) or triglyceride (TG) forms. Yet studies did not match the doses administered nor the concentrations of DHA and EPA per supplement across such comparisons, leading to questionable conclusions. This study was designed to compare the oral bioavailability of the same dose of both EPA and DHA in fish oil-EE vs. fish oil-TG vs. krill oil in plasma at the end of a four-week supplementation.

**Methods:**

Sixty-six healthy adults (n = 22/arm) were enrolled in a double blind, randomized, three-treatment, multi-dose, parallel study. Subjects were supplemented with a 1.3 g/d dose of EPA + DHA (approximately 816 mg/d EPA + 522 mg/d DHA, regardless of formulation) for 28 consecutive days, as either fish oil-EE, fish oil-TG or krill oil capsules (6 caps/day). Plasma and red blood cell (RBC) samples were collected at baseline (pre-dose on Day 1) and at 4, 8, 12, 48, 72, 336, and 672 h. Total plasma EPA + DHA levels at Week 4 (Hour 672) were measured as the primary endpoint.

**Results:**

No significant differences in total plasma EPA + DHA at 672 h were observed between fish oil-EE (mean = 90.9 ± 41 ug/mL), fish oil-TG (mean = 108 ± 40 ug/mL), and krill oil (mean = 118.5 ± 48 ug/mL), p = 0.052 and bioavailability differed by <24 % between the groups. Additionally, DHA + EPA levels were not significantly different in RBCs among the 3 formulations, p = 0.19, providing comparable omega-3 indexes.

**Conclusions:**

Similar plasma and RBC levels of EPA + DHA were achieved across fish oil and krill oil products when matched for dose, EPA, and DHA concentrations in this four week study, indicating comparable oral bioavailability irrespective of formulation.

**Trial registration:**

Clinicaltrials.gov identifier NCT02427373.

## Introduction

The positive benefits of long-chain n-3 polyunsaturated fatty acids (LC n-3-PUFA), docosahexaenoic acid (DHA) and eicosapentaenoic acid (EPA) for heart, brain and eye health are supported by many clinical studies [1–4]. However consumption of LC n-3-PUFA in the Western diet is low [5]. While no recommended daily intake for LC n-3-PUFA exists, authoritative bodies such as the Academy of Nutrition and Dietetics recommend 500 mg/d for cardiovascular health [6]. Intakes of LC n-3-PUFA can be increased by greater fish consumption or by taking dietary supplements, such as fish oil, algal oil or krill oil. Recently there has been conflicting and confusing information published on the relative oral bioavailability of these omega-3 supplements with a few studies suggesting that the phospholipid form (krill) is better absorbed than the ethyl ester (EE) or triglyceride (TG) forms of fish oils [7, 8]. Yet some studies did not match the doses administered, the number of capsules provided, and/or the amounts of DHA and EPA per supplement [7–11]. An open label study by Ulven, et al. [8] supplemented adults for seven weeks with nonequivalent doses of either krill oil (3 g/d) or fish oil TG (1.8 g/d) compared to a control (non- supplemented) group. The total EPA + DHA concentration and number of capsules administered were also not matched, making comparative bioavailability of these oils difficult. Schuchardt et al. [9] conducted an acute crossover bioavailability study comparing a single dose (1.68 g) of fish oil TG, EE and krill oil but the total concentration of each fatty acid, DHA and EPA was not equal between formulations. In order to reach a comparable total dose of EPA + DHA, subjects consumed 14 capsules of krill oil compared to 4 capsules of the fish oil. This was a single-dose kinetic study with the last sample collected at 72 h. The benefits of LC n-3-PUFA are based on chronic consumption. Therefore, it is important to assess levels of DHA and EPA after longer term use. Arterburn et al. [12] demonstrated that steady state plasma levels of LC n-3-PUFA were achieved after 4 weeks.

The objective of this double-blind, randomized, three-treatment, multi-dose, parallel study was to compare the relative oral bioavailability of a 1.3 g/d dose of DHA + EPA in the form of fish oil-EE vs. fish oil-TG vs. krill oil. The study was designed to test the hypothesis that total plasma levels of DHA + EPA would not be significantly different between the formulations at Week 4. Total dose of EPA and DHA was approximately 816 mg/d and 522 mg/d, respectively regardless of formulation. The primary outcome was to assess and compare the total plasma levels of EPA + DHA across all 3 treatments at the end of the 4-week (Hour 672) study.

## Methods

### Study population and study design

**Fig. 1:**
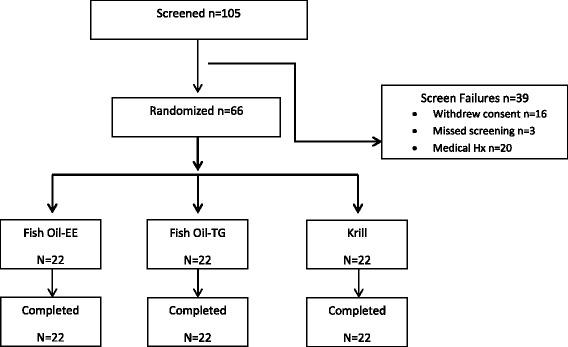
Disposition of subjects

Figure 1 provides a flow chart of subject disposition. One hundred-five healthy adults were recruited and screened via advertising and clinic database review. Sixty-six healthy adults were randomly assigned to one of the 3 formulations in the study. The study was approved by IntegReview IRB, Austin TX and was conducted in accordance with the clinical research guidelines as defined in the U.S. 21 CFR Parts 50, 56, and ICH E6, and the principles enunciated in the Declaration of Helsinki. All subjects provided written informed consent prior to undergoing any study-specific procedures. Major criteria for Inclusion: healthy male or females (not pregnant nor breastfeeding), 18–60 years of age (inclusive), with a body mass index between 18 and 30 kg/m^2^ (inclusive), and minimum weight of 50 kg (110 lb). Major exclusion criteria included a history or presence of diabetes, high triglycerides (≥240 mg/dL), or high cholesterol (≥240 mg/dL); history or presence of allergic or adverse response to omega-3-acids or sensitivity or allergy to fish or shellfish; history of coagulation disorder or current anticoagulation therapy; use of any nutritional supplements, omega-3 supplements, fish oil, chia, krill oil, or flaxseed within 3 months prior to the first dose of study product; consumed walnuts or any other food product supplemented with omega-3 fatty acids (e.g., orange juice) or fatty fish >2 times per week within 14 days prior to the first dose; history or presence of any major, clinically significant disease or condition. Subjects were admitted to the research unit 24 h prior to study product administration and remained at the unit until 12 h after the first dose on Day 1. Subjects continued daily dosing over 28 consecutive days (4 weeks). Plasma and red blood cell (RBC) samples were collected at baseline (pre-dose on Day 1), and at 4, 8, 12, 48, 72, 336, and 672 h after dosing for fatty acid analyses. Fasting blood draws were taken pre-dose and at hour 672. A standard high fat meal was administered prior to first dose of study product. Vital signs and clinical lab values (including lipids) were measured during and at the end of the study, respectively. Dietary intake of omega-3 fatty acids was reported on a food frequency questionnaire (FFQ) [13] at each in-clinic visit (for a total of 6 visits) and subjects were provided a list of foods that should not be consumed during the course of the study. Any reported adverse events (AEs) were recorded.

### Study products

All subjects consumed 6 capsules/day for 4 weeks. The krill oil product used (*Euphausia superba*) was commercially available NutriGold™ Double Strength Krill Gold oil (Neptune Technologies and Bioresources Inc, Laval, PQ). Krill capsules contained the antioxidant, astaxanthin, 840 μg/cap. Lipid class analysis of the krill oil capsule content as determined by TLC separation and fatty acid analysis of fractions indicated 44 % (w/w) phospholipid, 40 % triglyceride, 10 % diglyceride, 5 % sterol ester and 2 % free fatty acid (*DSM, internal analysis).* Fish oil-TG was commercially available Meg-3 1408 natural TG (DSM Nutritional Products, Mulgrave, NS). Fish oil-EE was Meg-3 1408 ethyl ester (DSM Nutritional Products, Mulgrave, NS). Fish oil capsules contained mixed natural tocopherol (1 mg/g) as antioxidant in the form of Tocoblend L70 IP (Vitablend, Wolvega, Netherlands) and paprika colorant RE04 (NutriCorp International, Windsor, ON) to match the krill oil. The following capsule fill weights and EPA + DHA content/capsule are listed below.

- Krill oil capsule fill weight = 1147 mg EPA = 135 mg/cap + DHA = 81 mg/cap
- Meg-3 1408 ethyl ester capsule fill weight = 1149 mg EPA = 140 mg/cap + DHA = 90 mg/cap
- Meg-3 1408 natural TG capsule fill weight = 1145 mg EPA = 140 mg/cap + DHA = 90 mg/cap

### Fatty acid analyses

Total lipids were extracted from 500 μL of packed RBCs using the method of Bligh and Dyer [14]. Plasma total lipids were extracted from 400 μL of plasma using the methods of Folch [15]. Tricosanoic free fatty acid (23:0) was added to each sample as an internal standard. All lipids were saponified with 0.5 N methanolic sodium hydroxide and the fatty acids were converted to methyl esters with 14 % BF_3_/methanol at 100 °C for 30 min [16]. Fatty acid methyl esters were analyzed by GLC using a Hewlett Packard 6890 equipped with a flame ionization detector. The fatty acid methyl esters were separated on a 30 m FAMEWAX capillary column (Restek, Bellefonte, PA; 0.25 mm diameter, 0.25 μm coating thickness) using hydrogen at a flow rate of 2.1 mL/min with a split ratio of 48:1. The chromatographic run parameters included an oven starting temperature of 130 °C that was increased at 6 °C/min to 225 °C, where it was held for 20 min before increasing to 250 °C at 15 °C/min, with a final hold of 5 min. The injector and detector temperatures were constant at 220 °C and 230 °C, respectively. Peaks were identified by comparison of retention times with external fatty acid methyl ester standard mixtures from NuCheck Prep (Elysian, MN).

### Statistical analyses

The primary outcome variable was the total plasma DHA + EPA concentration at Week 4 (Hour 672, fasted state). With an anticipated dropout rate of 10 %, it was expected that 60 completed subjects (approximately 20/group) would show no significant differences in total plasma DHA + EPA levels between the 3 formulations and would allow a determination of comparable bioavailability [10]. Differences between the plasma concentrations of DHA + EPA across the three nutritional supplements were determined by analysis of covariance (ANCOVA) modeling. The model included the pre-dose value as a covariate. The primary analysis was performed using the intent to treat population. The secondary analyses included comparisons of the concentrations of DHA + EPA in RBCs and concentrations of DHA and EPA separately in plasma and RBCs across nutritional supplements, using an analogous ANCOVA model; these analyses were performed using the intent to treat and per protocol populations. Analyses of demographic and screening variables were analyzed by ANOVA for between group differences of continuous variables or by chi-square tests for categorical variables. All tests of significance were performed at α = 0.05, 2-sided. As supportive evidence of comparable systemic exposure, concentrations of DHA + EPA, DHA, and EPA in plasma and RBCs were also compared across treatments in a pairwise manner, analogous to the analyses performed in bioequivalence assessments [17]. The two one-sided t-tests procedure was utilized to perform comparisons of fish oil-EE vs. fish oil-TG vs. krill oil levels at Week 4. The geometric mean ratios (GMR) and 90 % confidence intervals were presented for each comparison.

Study product compliance was measured by capsule counts and by the change in plasma EPA + DHA fatty acid levels from Hour 672 to pre-dose of ≥ 10 ug/mL. Changes less than 10 ug/mL or negative values were considered non-compliant.

All statistical analyses were conducted using SAS® (version 9.3, SAS Institute, Inc.).

## Results

### Subject characteristics

Sixty-six healthy adults (n = 22/arm) completed the study, overall mean ± SEM age = 35 (±1.4) years; 73 % were female, 59 % were Hispanic, mean weight = 68.7 (±1.4) kg, mean BMI = 25 (±0.3) kg/m^2^. Demographic characteristics of the 3 groups are shown in Table 1. No differences in any of these characteristics were found between the groups. Baseline vitals and ECG parameters were normal with no differences detected among the groups (data not shown). Dietary intake of omega-3 fatty acids, DHA, and EPA, as measured by the FFQ [13], showed low consumption at baseline, with an overall group mean ± SEM of DHA + EPA =89.5 (±7.2) mg/d; no significant differences between the groups were observed, p = 0.34. Over the course of the 4 week study, dietary intake of DHA + EPA declined to an average of 50–71 mg/d across the three groups, as assessed by the FFQ (p = 0.039). Pre-dose (baseline) total plasma mean (±SEM) DHA + EPA levels were not significantly different among the three groups (fish oil-EE 41.5 ± 3.3 ug/mL, fish oil-TG 42.7 ± 3.5 ug/mL, krill oil 37.5 ± 2.9 ug/mL, p = 0.51).

**Table 1:** Demographic Characteristics

		Fish or krill Oil: 1.3 g/d				
Parameter		Overall (N = 66)	Treatment A Ethyl Ester (N = 22)	Treatment B Triglycerides (N = 22)	Treatment C krill Oil (N = 22)	p-value*
Gender	Female	48 (72.7 %)	17 (77.3 %)	13 (59.1 %)	18 (81.8 %)	0.2011
	Male	18 (27.3 %)	5 (22.7 %)	9 (40.9 %)	4 (18.2 %)	
Age (years)	N	66	22	22	22	0.4523
	Mean (SEM)	35.45 (1.44)	37.41 (2.69)	35.95 (2.39)	33.00 (2.42)	
	Std Dev	11.72	12.64	11.21	11.37	
	Min, Max	19.00, 57.00	19.00, 57.00	19.00, 52.00	19.00, 55.00	
Ethnicity	Not Hispanic or Latino	27 (40.9 %)	10 (45.5 %)	9 (40.9 %)	8 (36.4 %)	0.8286
	Hispanic or Latino	39 (59.1 %)	12 (54.5 %)	13 (59.1 %)	14 (63.6 %)	
Race	Asian	1 (1.5 %)	1 (4.5 %)	0 (0.0 %)	0 (0.0 %)	0.6239
	Black or African American	7 (10.6 %)	2 (9.1 %)	3 (13.6 %)	2 (9.1 %)	
	White	57 (86.4 %)	19 (86.4 %)	18 (81.8 %)	20 (90.9 %)	
	Mixed: White, Black or African American	1 (1.5 %)	0 (0.0 %)	1 (4.5 %)	0 (0.0 %)	
Weight (kg)	N	66	22	22	22	0.3480
	Mean (SEM)	68.72 (1.43)	69.06 (2.83)	71.10 (2.55)	66.01 (1.96)	
	Std Dev	11.59	13.25	11.94	9.17	
	Min, Max	50.90, 111.20	50.90, 111.20	51.00, 89.00	52.80, 84.60	
Height (cm)	N	66	22	22	22	0.2408
	Mean (SEM)	164.35 (1.16)	164.98 (2.47)	166.39 (1.80)	161.68 (1.63)	
	Std Dev	9.45	11.59	8.44	7.66	
	Min, Max	149.50, 194.00	149.50, 194.00	153.00, 179.50	149.50, 181.00	
BMI (kg/m^2)	N	66	22	22	22	0.9056
	Mean (SEM)	25.32 (0.32)	25.22 (0.56)	25.53 (0.54)	25.22 (0.59)	
	Std Dev	2.60	2.62	2.54	2.75	
	Min, Max	19.80, 29.50	20.90, 29.50	20.80, 29.50	19.80, 29.20	

*P-values were derived by ANOVA for continuous variables and by the Chi-square test for categorical variables

Compliance with the study product was estimated at 100 % by capsule count and 93.9 % overall by change in plasma DHA + EPA levels (Hour_672_ -baseline ≥10 ug/mL). Four subjects (3 given fish oil-TG; 1 given krill oil) were removed in the per protocol analyses due to noncompliance with study product or consumption of foods supplemented with omega-3 fatty acids.

### Bioavailability of DHA + EPA in plasma, week 4

**Fig. 2:**
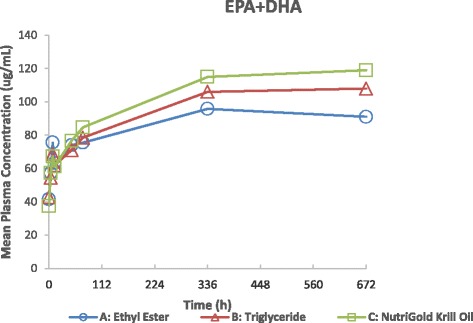
Mean Plasma DHA + EPA Concentration-Time Profiles after Administration of fish oil-Ethyl Ester, fish oil-Triglyceride, and krill oil, on a Linear Scale, Week 4 ANCOVA, p = 0.052, showing comparable bioavailability

Mean (±SD) DHA + EPA plasma concentrations at Week 4 (Fig. 2) were not significantly different between the three groups (90.99 ± 41.45 ug/mL fish oil-EE, 108.07 ± 40.41 ug/mL fish oil-TG, and 118.59 ± 48.74 ug/mL krill oil), p = 0.052, demonstrating similar bioavailability. Bioavailability of the three products differed by less than 24 %. As shown in Fig. 2, mean plasma concentrations at early time points (0–72 h) were also nearly identical between the groups. Pairwise comparisons of total DHA + EPA plasma concentrations at Week 4, using the two one-sided t-tests procedure, showed geometric mean ratios (90 % confidence intervals) as: 0.75 (0.59, 0.96) for fish Oil -EE vs. krill oil; 0.82 (0.64, 1.04) for fish oil-EE vs. fish oil-TG; and 0.92 (0.73, 1.15) for fish oil-TG vs. krill oil.

### Bioavailability of EPA or DHA in plasma, week 4

**Fig. 3:**
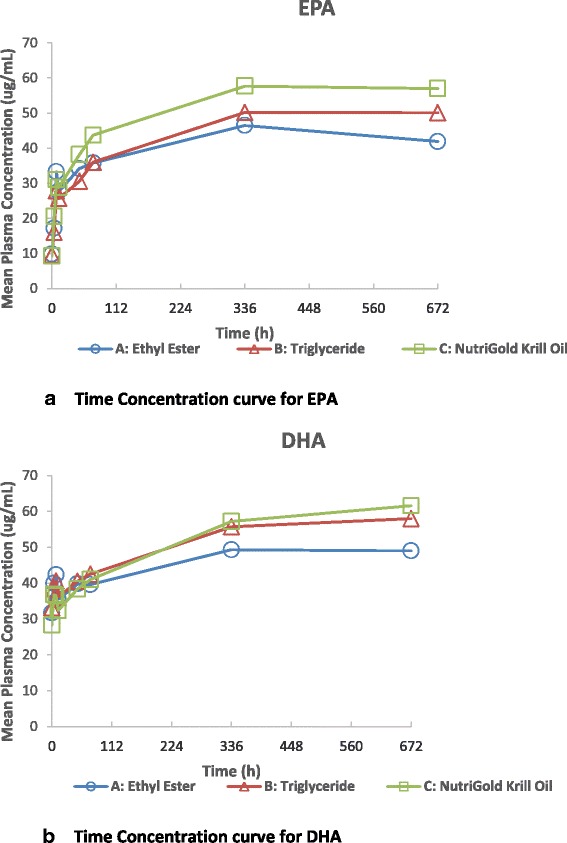
**a** Mean Plasma EPA Concentration-Time Profiles after Administration of fish oil-Ethyl Ester, fish oil-Triglyceride, and krill oil on a Linear Scale, Week 4 ANCOVA, p = 0.17, showing comparable bioavailability. **b** Mean Plasma DHA Concentration-Time Profiles after Administration of fish oil-Ethyl Ester, fish oil-Triglyceride, and krill oil on a Linear Scale. Week 4 ANCOVA, p = 0.013; a bioequivalence trend between fish oil-TG and krill oil is shown, GMR = 0.95 (0.79,1.13), p = 0.059

All three formulations had a higher EPA content relative to DHA, with an EPA:DHA ratio of approximately 1.6:1. Therefore, the bioavailability of each of these individual fatty acids was determined separately, in addition to the EPA + DHA previously presented. As shown in Fig. 3a, mean EPA plasma concentrations were similar at early time points following the administration of fish oil-EE, fish oil-TG, and krill oil. At Week 4, mean (±SD) EPA plasma concentrations were 41.95 ± 26.22 ug/mL for fish oil-EE, 50.07 ± 24.65 ug/mL for fish oil-TG, and 56.99 ± 30.40 ug/mL for krill oil, with no significant differences detected between groups, p = 0.17, indicating similar bioavailability. All pairwise comparisons of bioequivalence at Week 4 did not meet criteria.

Similarly, overall mean DHA plasma concentrations were comparable across the three groups. Differences were noted between 336 and 672 h, during which time the mean DHA plasma concentrations were similar for krill oil and fish oil-TG, while that for fish oil-EE was somewhat lower (Fig. 3b) and the Week 4 ANCOVA for the three groups was significant, p = 0.013. A trend of bioequivalence at Week 4 between fish oil-TG and krill oil was found with a GMR = 0.95 (0.79, 1.13), p = 0.059.

### Bioavailability of DHA + EPA and DHA or EPA alone in RBCs, week 4

**Fig. 4:**
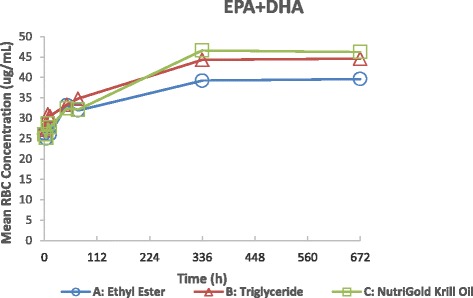
Mean Red Blood Cell DHA + EPA Concentration-Time Profiles after Administration of fish oil-Ethyl Ester, fish oil-Triglyceride, and krill oil on a Linear Scale, Week 4 ANCOVA, p = 0.19, showing comparable bioavailability

In RBCs, DHA + EPA levels were not significantly different between groups at Week 4 (fish oil-EE mean ± SD =39.6 ± 13 ug/mL; fish oil-TG =44.5 ± 12 ug/mL; krill oil =46.1 ± 13 ug/mL; p = 0.19, Fig. 4). The bioavailability in RBCs, as a proxy for long-term intake was comparable across treatments and differed overall by less than 14 %. Pairwise comparisons of fish oil-TG vs krill oil DHA + EPA RBC levels were significant with a GMR = 0.96 (0.82, 1.12) demonstrating comparable bioavailability. As seen in Fig. 4, pre-dose (baseline) DHA + EPA RBC levels were not significantly different between the three groups (fish oil-EE mean ± SEM = 26.3 ± 1.8 ug/mL, fish oil-TG = 27.6 ± 2.2 ug/mL, krill oil = 25.9 ± 1.7, p = 0.79). Analyses of mean (±SD) DHA RBC concentrations at Week 4 were not significantly different (27.68 ± 8.47 ug/mL for fish oil-EE, 30.59 ± 7.54 ug/mL for fish oil-TG, and 30.06 ± 7.37 ug/mL for krill, p = 0.48). Pairwise comparisons of fish oil-TG vs krill DHA RBC levels were significant with a GMR = 1.01 (0.89, 1.15) showing comparable bioavailability between these two products. While mean (±SD) EPA RBC concentrations at Week 4 among the groups were not significantly different (11.93 ± 6.04 ug/mL for fish oil-EE, 13.98 ± 5.64 ug/mL for fish oil-TG, and 16.11 ± 7.16 ug/mL for krill oil, p = 0.07), overall levels of EPA in RBCs were lower than DHA levels.

### Safety parameters

No clinically significant abnormalities or changes in vital signs or physical exams were observed over the course of the study. No significant changes in clinical laboratory tests, e.g. lipid panel parameters, glucose, or alkaline phosphatase, among others were noted. A total of 11 AEs were reported by 9 subjects (5 AEs in the krill oil group; 3 AEs in the fish oil-EE group; 3 AEs in the fish oil-TG group) over the course of the study. No serious AEs were reported. Table 2 shows the treatment–emergent AEs by verbatim term and group. The most commonly reported post-dose AEs were nausea (n = 2, both following fish oil-EE supplementation), and headache (n = 2: 1 following fish oil-TG supplementation and 1 following krill oil supplementation). Gastrointestinal AEs (e.g. abdominal cramps, bloating, gas) were minimal and were not different between the groups in this study. Of note, there were no instances of burping reported in any of the study groups.

**Table 2:** Treatment-emergent adverse events by first occurrence^a^ and verbatim term safety population

	Fish or krill Oil: 1.3 g/d		
Verbatim term	Treatment A Ethyl Ester (N = 22)	Treatment B Triglycerides (N = 22)	Treatment C krill Oil (N = 22)
Abdominal bloating	0 (0.0 %)	1 (4.5 %)	0 (0.0 %)
Abdominal cramps	0 (0.0 %)	1 (4.5 %)	0 (0.0 %)
Abdominal pain	0 (0.0 %)	0 (0.0 %)	1 (4.5 %)
Hair loss	0 (0.0 %)	0 (0.0 %)	1 (4.5 %)
Headache	0 (0.0 %)	1 (4.5 %)	1 (4.5 %)
Insect bite	1 (4.5 %)	0 (0.0 %)	0 (0.0 %)
Intermittent epistaxis	0 (0.0 %)	0 (0.0 %)	1 (4.5 %)
Intermittent vasovagal	0 (0.0 %)	0 (0.0 %)	1 (4.5 %)
Nausea	2 (9.1 %)	0 (0.0 %)	0 (0.0 %)
Total events	3	3	5

^a^Subjects may have had more than one adverse event. For these subjects, the first occurrence of the AE was tabulated

## Discussion

No significant differences in mean fasting plasma concentrations of DHA + EPA, as a proxy for steady-state LC n-3-PUFA levels, were found after four weeks of supplementation of a 1.3 g/d dose of DHA + EPA in fish oil-EE, or fish oil-TG or krill oil, demonstrating comparable oral bioavailability (i.e. amount of active ingredient absorbed). The results demonstrate <24 % difference in bioavailability among the 3 products. The four week time course of supplementation was chosen as this time period typically demonstrates that omega-3 fatty acid plasma levels reach plateau (as observed in Figs. 2, 3a, b and 4) in healthy adults and is an estimate of steady-state levels and representative of short term intake. Our results also show nearly identical bioavailability among the groups at early timepoints (<48 h) of the plasma time course, illustrating no better bioavailability of one product versus another when equivalent concentrations of omega-3 fatty acids are administered. In contrast, a study by Kagan et al. [18] showed significantly increased plasma concentrations of EPA from an algal oil supplement compared to krill oil at 5–10 h post-supplementation with greater peak and total systemic exposure parameters, likely reflecting the greater EPA content of the algal product. Another single-dose, acute bioavailability study very recently published [19] found higher plasma phospholipid EPA and DHA levels at 72 h in krill oil vs. krill meal or fish oil, but did not find higher levels between the krill meal and fish oil, even given the same dose with relatively equal amounts of DHA and EPA. As Kohler [19] pointed out, this result argues against an interpretation that phospholipids are better absorbed than triglycerides and suggested that a longer term supplementation study, measuring RBC levels, was needed. As phospholipid levels represent only one fraction of the total fatty acid profile, it may also be important to measure changes in the other lipid fractions or measure total plasma and erythrocyte fatty acid changes over time.

No significant differences in mean RBC concentrations of DHA + EPA at week 4 were obtained between our three formulations, again demonstrating similar bioavailability. This is important as it shows an integrated picture of DHA+ EPA utilization in the body and RBC levels are a proxy for long-term bioavailability. The DHA + EPA percent of total RBC fatty acids, the omega-3 index, is an important cardiac risk factor and our study demonstrates that all these products provide adequate DHA + EPA levels to achieve this benefit [20]. The geometric mean ratio for DHA + EPA comparing fish oil-TG vs krill oil in RBCs at Week 4 were significant, indicating comparable bioavailability between these two products. For RBC DHA levels only, all three oil formulations were equally bioavailable. Since RBC fatty acid content is importantly related to tissue fatty acid status, these results suggest no differences in bioavailability in tissues among the fish oil formulations and krill oil in this 4 week study.

Examination of the individual fatty acids, DHA and EPA each showed comparable bioavailability in plasma levels at Week 4 among the fish oil and krill oil products. However, DHA concentrations at hours 336 and 672 were lower in the fish oil-EE group compared to fish oil-TG or krill oil groups, as seen in Fig. 3b. Other studies have indicated lower plasma levels of DHA and/or EPA with fish oil-EE administration versus re-esterified TG [9, 21, 22], suggesting lower bioavailability and reduced functional outcomes, e.g. triacylglycerol lowering with the EE form. However not all studies have shown this effect and the marketed product approved for hypertriglyceridemia, *Lovaza™* is a fish oil-EE formulation that significantly reduces triacylglycerol.

Our study demonstrated comparable bioavailability among the 3 formulations, however variability and wider confidence intervals were observed, especially in the plasma levels. This variability likely impacted the bioequivalence assessments among the groups and may be due to inter-individual variability and the smaller sample size in this parallel study. A larger study or one with a cross-over design may further elucidate these findings.

Different results observed in other omega-3 studies using krill oil and fish oil may be due to non-equivalent doses or unmatched DHA and EPA concentrations, non-compliance, especially when large numbers of capsules were used, or confounders such as increased dietary intake of omega-3 fatty acids [11, 23]. The compliance in our study was over 90 % (measured by plasma fatty acids levels and capsule counts) and dietary intake of omega-3s was low in this study sample. The formulations were well tolerated and no differences in gastrointestinal symptoms, of which there were very few, were noted. It is possible that the phospholipid content in krill oil may affect its bioavailability, however krill oil composition in various commercial products varies widely in phospholipid content from approximately 19–81 % [24] and this has not been systematically examined in longer-term dosing trials. The krill oil used in our study contained 43.7 % phospholipid (not a minor component) and demonstrated similar bioavailability to both TG and EE fish oil forms.

In conclusion, our results demonstrate similar plasma and RBC levels of EPA + DHA were achieved with fish oil and krill oil products when matched for dose and EPA + DHA content in this four week study, indicating comparable oral bioavailability irrespective of formulation.

## References

[1] Nestel P, Clifton P, Colquhoun D, Noakes M, Mori T, Sullivan D (2015). Indications for Omega-3 Long Chain 3 Polyunsaturated Fatty Acid in the Prevention and Treatment of Cardiovascular Disease. Heart Lung Circ.

[2] Abu-Ouf N, Jan M (2014). The Influence of Fish Oil on Neurological Development and Function. Can J Neurol Sci..

[3] Yurko-Mauro K, Alexander D, van Elswyk M (2015). Docosahexaenoic Acid and Adult Memory: A Systematic Review and Meta-Analysis. PLoS ONE.

[4] Dawczynski J, Jentsch S, Schweitzer D, Hammer M, Lang G, Strobel J (2013). Long term effects of lutein, zeaxanthin and omega-3-LCPUFAs supplementation on optical density of macular pigment in AMD patients: the LUTEGA study. Graefes Arch Clin Exp Ophthalmol.

[5] U.S. Department of Agriculture, Agricultural Research Service (2012). Nutrient Intakes from Food: Mean Amounts Consumed per Individual, by Gender and Age, What We Eat in America, NHANES 2009–2010.

[6] http://www.eatright.org/resource/health/wellness/heart-and-cardiovascular-health/heart-health-anddiet. Accessed 7/13/15.

[7] Ramprasath V, Eyal I, Zchut S, Jones PJH (2013). Enhanced increase of omega-3 index in healthy individuals with response to 4-week fatty acid supplementation from Krill oil versus fish oil. Lipids Health Dis.

[8] Ulven S, Kirkhus B, Lamglait A, Basu S, Elind E, Haider T (2011). Metabolic effects of Krill oil are essentially similar to those of Fish oil but at lower dose of EPA and DHA, in healthy volunteers. Lipids.

[9] Schuchardt JP, Schneider I, Meyer H, Neubronner J, von Schacky C, Hahn A (2011). Incorporation of EPA and DHA into plasma phospholipids in response to different omega-3 fatty acid formulations - a comparative bioavailability study of fish oil vs. Krill oil. Lipids Health Dis.

[10] Maki KC, Reeves MS, Farmer M, Griinari M, Berge K, Vik H (2009). Krill oil supplementation increases plasma concentrations of eicosapentaenoic and docosahexaenoic acids in overweight and obese men and women. Nutr. Res..

[11] Salem N, Kuratko C (2014). A reexamination of Krill bioavailability studies. Lipids Health Dis.

[12] Arterburn L, Hall EB, Oken H (2006). Distribution, interconversion, and dose response of n-3 fatty acids in humans. Am J Clin Nutr.

[13] Kuratko C (2013). Food-frequency questionnaire for assessing long-chain u-3 fatty-acid intake, Re: Assessing long-chain ω-3polyunsaturated fatty acids: A tailored food-frequency questionnaire is better. Nutrition.

[14] Bligh EG, Dyer WJ (1959). A rapid method of total lipid extraction and purification. Can. J. Biochem. Physiol..

[15] Folch J, Lees M, Sloane S (1957). A simple method for the isolation and purification of total lipids from animal tissue. J Biol Chem..

[16] Morrison WR, Smith LM (1964). Preparation of fatty acid methyl esters and dimethylacetals from lipids with boron fluoride-methanol. J Lipid Res..

[17] Food US, Administration D (2003). Guidance for Industry: Bioavailability and Bioequivalence Studies for Orally Administered Drug Products—General Considerations.

[18] Kagan M, West A, Zante C, Calder P (2013). Acute appearance of fatty acids in human plasma – a comparative study between polar-lipid rich oil from the microalgae Nannochloropsisoculata and Krill oil in healthy young males. Lipids Health Dis.

[19] Kohler A, Sarkinnen E, Tapola N, Niskanen T, Bruheim I (2015). Bioavailability of fatty acids from krill oil, krill meal and fish oil in healthy subjects: a randomized, single-dose, crossover trial. Lipids Health Dis.

[20] Harris WS (2008). The Omega-3 Index as a risk factor for Coronary Heart Disease. Am J Clin Nutr..

[21] Schuchardt JP, Neubronner J, Kressel G, Merkel M, von Schacky C, Hahn A (2011). Moderate doses of EPA and DHA from re-esterified triacylglycerols but not from ethyl-esters lower fasting serum triacylglycerols in statin-treated dyslipidemic subjects: Results from a six month randomized controlled trial. PLEFA.

[22] Dyerberg J, Madsen P, Møller JM, Aardestrup I, Schmidt EB (2010). Bioavailability of marine n-3 fatty acid formulations. PLEFA.

[23] Laidlaw M, Cockerline C, Rowe W (2014). A randomized clinical trial to determine the efficacy of manufacturers’ recommended doses of omega-3 fatty acids from different sources in facilitating cardiovascular disease risk reduction. Lipids Health Dis.

[24] Araujo P, Zhu H, Breivik JF, Hjelle JI, Zeng Y (2014). Determination and structural elucidation of triacylglycerols in krill oil by chromatographic techniques. Lipids.

